# Structure-based virtual screening and molecular dynamics studies to explore potential natural inhibitors against 3C protease of foot-and-mouth disease virus

**DOI:** 10.3389/fvets.2023.1340126

**Published:** 2024-01-17

**Authors:** Sthitaprajna Sahoo, Hak-Kyo Lee, Donghyun Shin

**Affiliations:** ^1^Department of Agricultural Convergence Technology, Jeonbuk National University, Jeonju, Republic of Korea; ^2^Department of Animal Biotechnology, Jeonbuk National University, Jeonju, Republic of Korea

**Keywords:** virtual screening, MD simulation, FMDV, DFT, 3C protease

## Abstract

Foot-and-mouth disease (FMD) is a highly infectious animal disease caused by foot-and-mouth disease virus (FMDV) and primarily infects cloven-hoofed animals such as cattle, sheep, goats, and pigs. It has become a significant health concern in global livestock industries because of diverse serotypes, high mutation rates, and contagious nature. There is no specific antiviral treatment available for FMD. Hence, based on the importance of 3C protease in FMDV viral replication and pathogenesis, we have employed a structure-based virtual screening method by targeting 3C protease with a natural compounds dataset (*n* = 69,040) from the InterBioScreen database. Virtual screening results identified five potential compounds, STOCK1N-62634, STOCK1N-96109, STOCK1N-94672, STOCK1N-89819, and STOCK1N-80570, with a binding affinity of −9.576 kcal/mol, −8.1 kcal/mol, −7.744 kcal/mol, −7.647 kcal/mol, and − 7.778 kcal/mol, respectively. The compounds were further validated through physiochemical properties and density functional theory (DFT). Subsequently, the comparative 300-ns MD simulation of all five complexes exhibited overall structural stability from various MD analyses such as root mean square deviation (RMSD), root mean square fluctuation (RMSF), radius of gyration (Rg), solvent accessible surface area (SASA), H-bonds, principal component analysis (PCA), and free energy landscape (FEL). Furthermore, MM-PBSA calculation suggests that all five compounds, particularly STOCK1N-62634, STOCK1N-96109, and STOCK1N-94672, can be considered as potential inhibitors because of their strong binding affinity toward 3C protease. Thus, we hope that these identified compounds can be studied extensively to develop natural therapeutics for the better management of FMD.

## Introduction

Foot-and-mouth disease (FMD) is a highly infectious disease that significantly affects global livestock production by affecting cloven-hoofed animals such as cattle, pigs, sheep, goats, and deer (1). FMD outbreaks have occurred in various parts of the world over the years. These outbreaks can have significant economic and agricultural impacts due to trade restrictions imposed on countries affected by FMD (2). Due to the highly infectious nature of the virus and the heightened international trade in animals and animal products, it is interesting to note that no nation is entirely safe from the threat. The World Organization for Animal Health (WOAH) urges global cooperation in addition to independent regional initiatives to combat the disease successfully. The disease is caused by a single-stranded positive RNA virus named foot and mouth disease virus (FMDV), which belongs to the Picornaviridae family and has seven serotypes (O, A, C, Asia1, and SAT1-3) and numerous strains, making it a challenging disease to control and eradicate (3). In most FMD outbreak regions, including Asia, the Middle East, India, Africa, and South America, the O serotype is prevalent (4). The symptoms of FMDV can vary depending on the species and age of the affected animal as well as the serotype and strain of the virus. Generally, severe symptoms are noticed in cattle and reared swine, and they exhibit clinical symptoms 24 to 48 h after FMDV infection. At this point, high viral counts can be observed in the serum of infected animals. Fever, shivering, salivary drooling, and the development of blisters or vesicles on the epithelium of the tongue, nose, coronary bands, and teats are the hallmarks of FMD (5).

The treatment of FMDV primarily focuses on controlling the spread of the virus and managing the clinical symptoms in affected animals. Vaccination against FMDV was vital in maintaining and preventing most FMD outbreaks (6). Countries adopt different vaccination strategies based on the prevailing FMDV strains and epidemiological situations where vaccine-containing inactivated FMDV strains were commonly used (2, 7). Although FMDV vaccines can reduce some FMD outbreaks, vaccination against FMDV does not prevent the transmission of the virus and takes several days to trigger an immune response against FMDV. Additionally, it might be challenging to match the virus vaccine to the multiple serotypes and subtypes. To significantly cope with the genetic variability and reduce clinical signs, the ideal antiviral treatments should be instantly and widely active on diverse virus serotypes. However, it is essential to note that no specific antiviral therapy is available for FMDV, and most efforts are directed toward prevention, vaccination, and supportive care; thus, antiviral therapeutics are still required for quick prevention and management of FMD spread.

**Figure 1:**
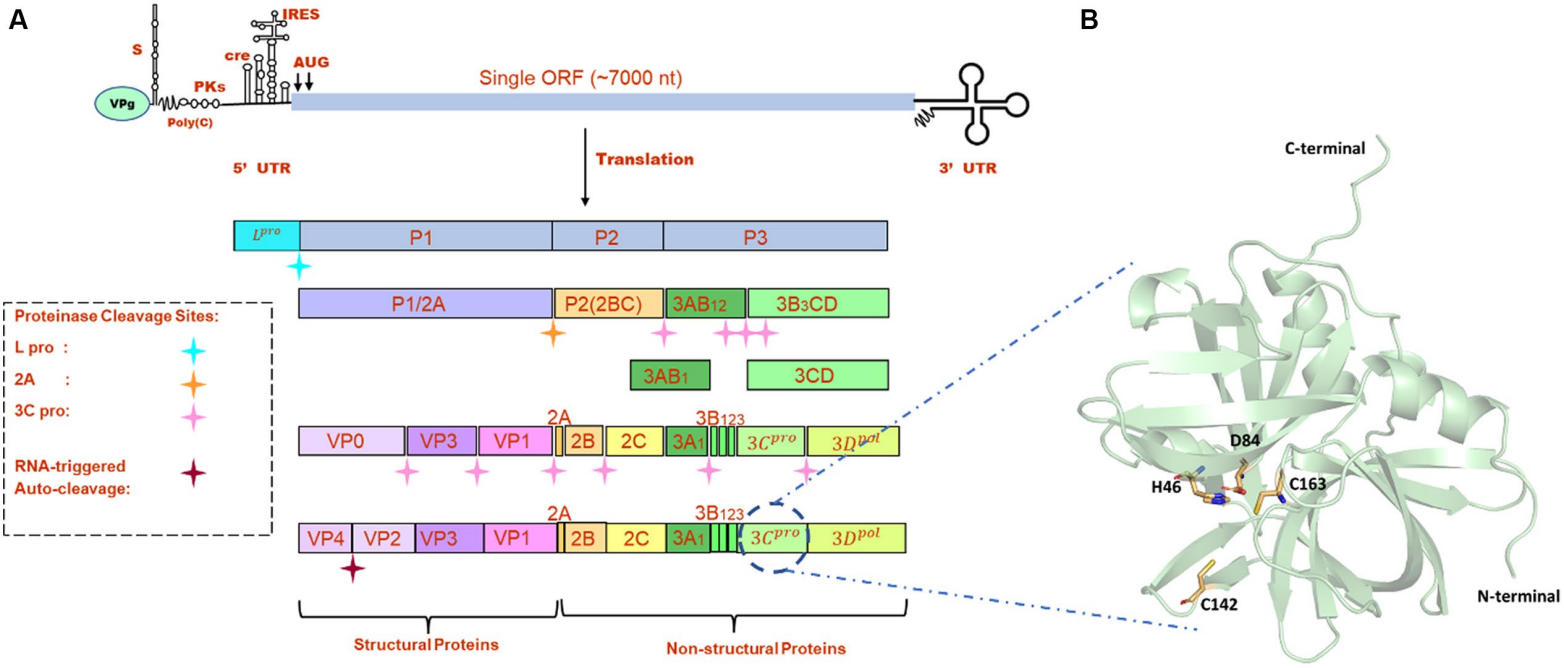
**(A)** Genome organization FMDV. **(B)** The modeled structure of 3C protease is represented in a pale green cartoon structure where catalytic residues (C163, H46, and D84), and active site residue C142 is represented in the stick model.

FMDV is a small, non-enveloped, spherical virus belonging to the family of Picornaviridae, having a diameter of 25–30 nm. FMDV consists of a single-stranded +ve sense RNA genome having 8,400 nucleotides (nt), which includes 5’UTR (1,300 nt), open-reading frame (ORF) (7,000 nt), and 3’UTR (90 nt) (1). This long ORF of the viral genome encodes a single large polyprotein, which then undergoes proteolytic processing to form 4 structural and 10 non-structural proteins (Figure 1A). Primary co-translation produces four primary individual products, namely, Lpro, P1/2A, P2, and P3. P1/2A is further processed to form four structural proteins, i.e., VP4, VP2, VP3, and VP1. Lpro is a papain-like protease that releases itself from the polyprotein by cleaving its own C-terminal and N-terminal of VP4. The P2 and P3 portions of the polyprotein were further processed to form mature individual proteins, i.e., 2A, 2B, 2C, 3A, 3B_1_, 3B_2_, 3B_3_, 3Cpro, and 3Dpol (8). Upon entry into receptive host cells, the viral genome is immediately translated into a long polyprotein precursor that must then be broken down by virally encoded protease to produce the 14 individual protein products that are needed for RNA replication and for the construction of new viral particles (9). One of the most highly conserved genes in the viral genome, FMDV 3C protease (3Cpro), is the key component of the aforementioned proteolytic activity and is responsible for 10 of the 13 cleavages. It is a trypsin-like serine protease that also cleaves the host proteins present in the infected cells such as eIF4G (10). Additionally, during FMDV infection, FMDV 3Cpro cleaves histone H3, causing a generalized repression of cellular transcription (11). The crucial role of 3Cpro in the viral replication process and cleaving activity makes it a possible antiviral drug target that can be combined with other strategies to prevent and control FMDV. There are antiviral medications available that target the picornavirus protease. For instance, rupintrivir, an inhibitor of human rhinovirus (HRV) 3C protease, demonstrated strong antiviral activity in cultured cells. However, the effectiveness of rupintrivir against a natural HRV infection was insignificant (12, 13).

Research on natural compounds as antiviral therapeutics has gained considerable attention due to the potential benefits of these compounds, including their broad-spectrum activity, low toxicity, and diverse mechanisms of action (14). Many natural compounds isolated from different natural resources such as plants, microorganisms, and marine species have exhibited inhibitory activity against a wide range of pathogens and viruses (15, 16). Since the extraction of natural compounds and antiviral activity evaluation require funding as well as specialized experimental equipment, exploring potent FMDV inhibitors from natural sources is challenging. This strategy can also be integrated with molecular biological approaches to uncover the novel mechanisms of inhibition using structural relationships. In recent years, the integration of structure-based virtual screening (SBVS), MD simulations, and density functional theory (DFT) analysis enhances the drug discovery process by providing a multi-faceted understanding of the molecular interactions between drugs and their targets. This integrated approach enables more informed decision-making in the identification, optimization, and design of novel drug candidates (17–21). Hence, in the current study, we explored potential natural compounds against FMDV 3Cpro through structure-based virtual screening. Moreover, we have also employed a molecular dynamics (MD) approach to analyze the binding affinities and detailed structural interaction of the selected inhibitors with the targeted protein.

## Theory and methods

### Preparation of target protein

All the crystal structures of FMDV 3Cpro that were available in the RCSB Protein Data Bank^1^ contained mutations either at active site residues (C142L and C142S) or catalytic residues (C163A and D84E) (22). As serotype O was prevalent in most of the FMD outbreaks, we retrieved the full-length amino acid sequence (213 amino acids) of FMDV 3Cpro of O serotype from UniProt database (Uniprot ID: P03305)^2^ and modeled the 3D structure of wild type 3Cpro using the most advanced deep learning model AlphaFold2, where structures having PDB ID: 2BHG, 2 J92, 2WV4, and 5HM2 were used as template (Figure 1B) (23). We have also validated the modeled structure of FMDV 3C pro using the Ramachandran plot (Supplementary Figure S1). AlphaFold2 is an advanced deep learning model developed by DeepMind that specializes in protein folding prediction. While it can be used for a variety of protein structure prediction tasks, including *de novo* folding, one of its significant applications is in homology modeling. The protein preparation wizard of Schrodinger was used to prepare the target protein structure where missing atoms were incorporated, ionization states were generated, and proper bond orders were assigned for optimal docking analysis (24).

### Selection of natural compounds

We retrieved 69,040 compounds from the natural compound dataset of the InterBioScreen database.^3^ The InterBioScreen database is a comprehensive collection of natural compounds and small molecules that are of interest in drug discovery and medicinal chemistry research. It contains a vast array of compounds that come from naturally occurring substances, such as plants, microorganisms, marine organisms, and other biological materials, that assist researchers in the identification and selection of potential lead compounds for drug development. All the natural compounds from this database were downloaded in a structured data file format and considered for virtual screening.

### Structure-based virtual screening

Structure-based virtual screening (SBVS) is a computational technique used in drug discovery to identify and prioritize potential drug candidates from large compound libraries or databases with a certain molecular target (25). It involves the use of computer algorithms and molecular modeling methods to predict the binding affinity and biological activity of compounds toward a target protein or biological target along with their drug-like properties using Lipinski’s rule of five. Glide and virtual screening module of Schrodinger was used to perform molecular docking in three stages, i.e., high throughput virtual screening (HTVS), standard precision (SP), and extra precision (XP) using OPLS3 force field (26–28). Prior to the molecular docking, a grid box was generated using the catalytic and binding site residues such as C142, S182, D84, C163, and H46 of FMDV 3C protease (29–31). The interaction of the top five screened compounds was visualized using Maestro, Discovery studio, and PyMOL to determine the crucial amino acid residues that were involved in protein-ligand interaction (32, 33).

### Density functional theory calculation

Geometry optimization of the selected five compounds was performed through DFT using the DMOL3+ server in BIOVIA Material Studio version 2017 R2. Generalized gradient approximation (GGA) and Perdew–Burke–Ernzerhof (PBE) functions with double numerical plus polarization (DNP) basis set were chosen to calculate the highest occupied molecular orbital (HOMO), lowest unoccupied molecular orbital (LUMO), energy gap, and electronic chemical potential (μ) (34). The maximum force, energy convergence accuracy, gradient convergence, maximum displacement, displacement convergence, and optimal iterations were set to be 0.004 Ha/A, 2 × 10^−5^ Ha, 4 × 10^−3^ Å, 0.3 Å, 5 × 10^−3^ Å, and 50, respectively.

### Molecular dynamics simulation

Molecular dynamics (MD) simulations of the top five screened protein-ligand complexes were performed using the GROMACS 2022.2 package and CHARMM36 force field to analyze the conformational changes and dynamic behavior at the atomistic level (35–37). The ligand topology of the selected five compounds was generated using the CGenFF webserver, and Na+/Cl- counter ions were added to neutralize the complex structures (38). The system was further solvated using a TIP3P water model and a dodecahedron periodic boundary condition. The steepest-descent algorithm was used to minimize the system’s energy followed by conjugate gradient (39). The particle Mesh Ewald (PME) method was used to compute the long-range electrostatic interactions, and the LINear Constraint Solver LINC0 algorithm was used to calculate the Lennard-Jones and Coulomb interactions with a radius of 10 Å cutoff distance (40–42). The temperature coupling was maintained at 300 K using the Berendsen thermostat (V-rescale) coupling algorithm, while pressure coupling was maintained at 1 Pascal bar using the Parrinello–Rahman pressure coupling algorithm (43, 44). After energy minimization, the system was equilibrated for 100 ps each under NVT and NPT conditions to retain the volume, pressure, and temperature. All five systems were subjected to 300 ns MD production run and coordinates were saved at a consistent time step of 2 fs intervals. From the final trajectory information, we studied the root mean square deviation (RMSD), radius of gyration (Rg), root mean square fluctuation (RMSF), solvent accessible surface area (SASA), and H-bonds analysis using gromac’s modules. The global motion of all complexes was also analyzed with principal component analysis (PCA) considering the last 20 ns trajectory information, and free energy landscape (FEL) was studied from the first two principal components (PCs) using the gmx sham tool, as reported in our previous study (45, 46).

### Binding free energy calculation

Molecular mechanics/Poisson–Boltzmann surface area (MM-PBSA) is a computational method used to estimate the binding free energies of biomolecular complexes such as protein–ligand, protein–DNA, or protein–protein complexes (47). It combines molecular mechanics force fields, continuum solvent models, and empirical solvation energy terms to estimate the thermodynamic properties of protein–ligand interactions. MM-PBSA also allows the decomposition of the free energy of binding into different components, such as van der Waals interactions, electrostatic interactions, solvation-free energy, and entropy contributions. This decomposition helps in understanding the individual energetic contributions of different molecular interactions that allow researchers to focus on specific aspects of ligand optimization, such as improving hydrogen bonding, reducing steric clashes, or enhancing electrostatic interactions. MM-PBSA calculations are commonly used in computer-aided drug design and lead optimization studies to evaluate the binding affinities of ligands, compare different ligand poses, and guide the selection of promising drug candidates (48). MM-PBSA results can be compared with experimental binding affinities to validate the accuracy of the computational predictions. This validation is essential for building confidence in the computational approach and its applicability in drug discovery. Mathematically, binding free energy (ΔG_bind_) can be represented as follows:


$$\Delta G_{\text{bind}}=\Delta G_{\text{mm}}+\Delta G_{\text{ps}}+\Delta G_{nps}-T\Delta S$$

Here, ΔG_mm_ represents the molecular mechanics energy by considering van der Waals and electrostatic interactions. ΔG_ps_ refers to polar solvation energy, and ΔG_nps_ refers to non-polar solvation energy. TΔS represents total entropic contribution, where T and S are denoted as temperature and entropy, respectively. The binding-free energy of the selected five compounds was calculated with respect to the 3C protease protein from the last 200 ns MD trajectory using the gmx_MMPBSA tool (49, 50).

## Results

### Identification of potent inhibitors through SBVS

Structure-based virtual screening (SBVS) in drug discovery offers several advantages, including the ability to screen a large number of compounds efficiently, reduced cost and time compared to experimental screening, and the potential to explore a wide chemical space. In the current study, we have considered the natural compounds subset from the InterBioScreen database (n = 69,040) against FMDV 3C protease. Initially, the large natural compounds dataset was screened with the Qikprop module, following Lipinski’s Ro5 filtering, where 48,952 compounds were screened. The high throughput virtual screening module screened 4,895 compounds, and 2,695 natural compounds were filtered for SP docking. In total, 269 compounds were pulled down from SP, and finally, 26 compounds were considered for XP docking for more accurate screening. Subsequently, the top five protein-ligand complexes with maximum glide score (−9.576 to −7.778 kcal/mol) were considered for further analysis (Table 1).

**Table 1:** Binding affinity of the top five screened compounds along with the amino acid residues involved in interactions.

Sl. No.	Compound ID	Docking score (kcal/mol)	XP GScore (kcal/mol)	H-bond interactions
1.	STOCK1N-62634	−9.575	−9.576	H46, D146, D144, G184
2.	STOCK1N-96109	−7.878	−8.1	E50, D146, M143, G184
3.	STOCK1N-94672	−7.674	−7.744	H46, G161
4.	STOCK1N-89819	−7.451	−7.647	M143, G184
5.	STOCK1N-80570	−7.34	−7.778	M143, H181, G184, N186, Y190

**Figure 2:**
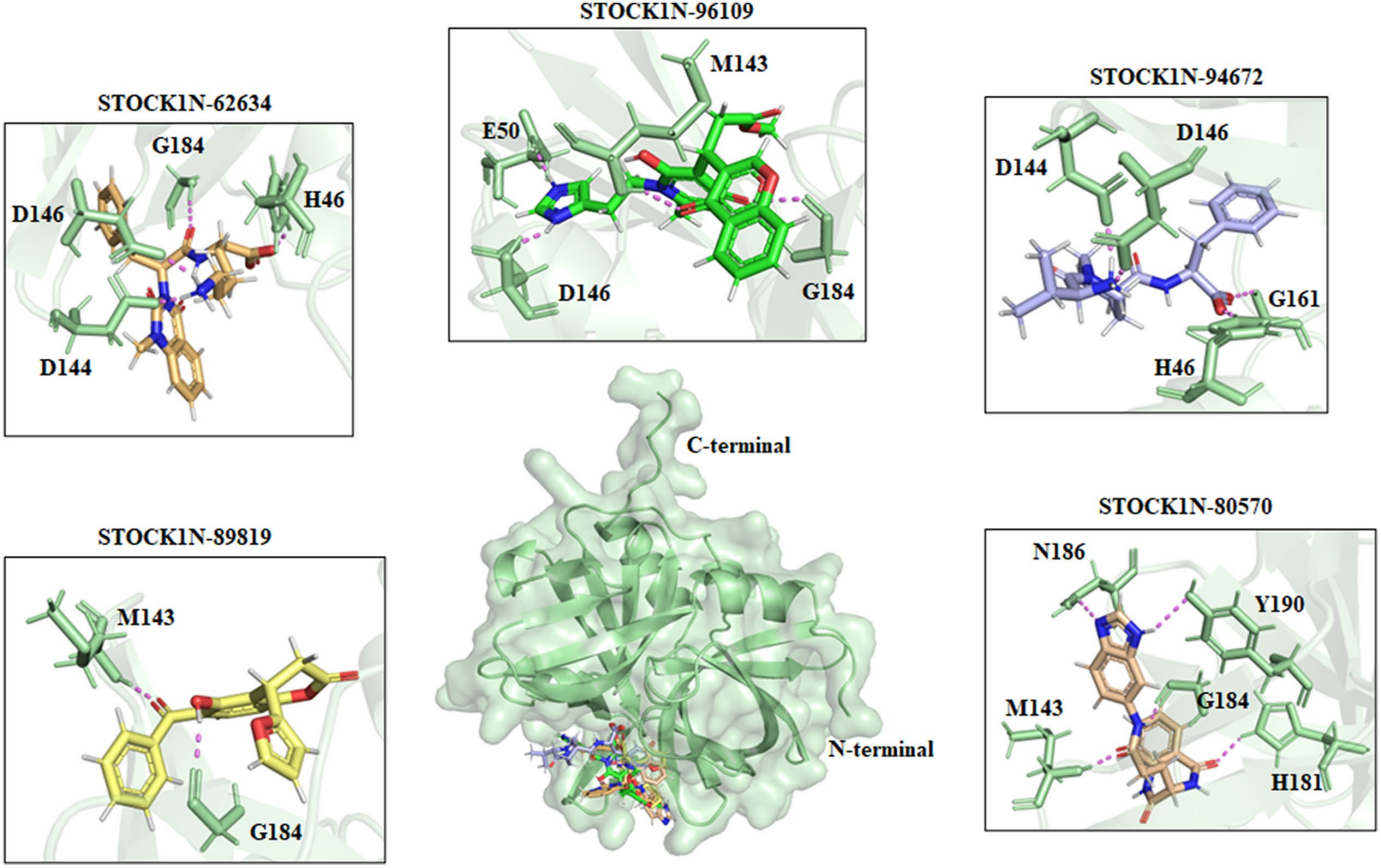
Interaction of the top five screened compounds (STOCK1N-62634, STOCK1N-96109, STOCK1N-94672, STOCK1N-89819, and STOCK1N-80570) with the 3C protease protein. Interacting residues of the protein were labeled in the pale green stick model, and interactions were represented in a violet dashed line.

All the top five screened compounds such as STOCK1N-62634, STOCK1N-96109, STOCK1N-94672, STOCK1N-89819, and STOCK1N-80570 exhibited the highest binding affinity of −9.576, −8.1, −7.744, −7.647, and − 7.778 kcal/mol, respectively. STOCK1N-62634 exhibited the highest binding affinity of −9.576 kcal/mol by forming four H-bond interactions with H46, D146, D144, and G184 and two pi-alkyl interactions with M143 and A183. Similarly, compound STOCK1N-96109 exhibited the binding affinity of −8.1 kcal/mol by forming four hydrogen bond interactions with E50, D146, M143, and G184 and one pi-cation interaction with the active site residue H46 (Figure 2). Despite having two H-bond interactions with 3C protease, compounds STOCK1N-94672 and STOCK1N-89819 exhibited a strong binding affinity. The 2D structures of the screened compounds and their 2D molecular interaction profile with the 3C protease protein are shown in Supplementary Figure S2.

### Assessment of drug-likeness through physiochemical properties

The pharmacokinetics and physiochemical properties of all the five screened compounds were evaluated using the QikProp module of Schrodinger. To acquire a safe side and potentially optimal drug-like behavior, a compound needs to fulfill at least four of the five characteristics of Lipinski’s rule of five, i.e., molecular weight < 500 Daltons, ≤5 hydrogen bond donors, and ≤ 10 hydrogen bond acceptors, lipophilicity <5, and molar refractivity between 40 and 130. The physiochemical and drug-likeness properties were evaluated and mentioned in Table 2. The ADME prediction results revealed that all the selected five natural compounds have an acceptable number of H-bond donors and acceptor, molecular weight, and lipophilicity with no violation of Lipinski’s rule of five. Hence, all these compounds can be considered as ideal drug candidates.

**Table 2:** Predicted ADME properties for top hit lead compounds.

Compounds	MW	H-bond donors	H-bond acceptors	QPlogPo/w	QPlogHERG	Rule of five violations
STOCK1N-62634	452.509	3	8	0.785	−3.814	Nil
STOCK1N-96109	449.462	2	8	3.145	−6.008	Nil
STOCK1N-94672	375.467	3	7	−0.471	−1.487	Nil
STOCK1N-89819	334.328	0	4	3.210	−5.726	Nil
STOCK1N-80570	363.375	4	9	0.930	−5.992	Nil

#MW: Molecular weight. #QPlogPo/w: Predicted octanol/water partition coefficient. #QPlogHERG: Predicted IC50 value for blockage of the HERG K+ channels.

### Quantum mechanics

The quantum mechanics studies were performed using geometry optimization-based DFT calculations. DFT analysis results revealed energy values of HOMO, LUMO, energy gap, and electronic chemical potential (μ) and are shown in Table 3. HOMO values represent the electrophilic attack site by calculating its potential to distribute and donate electrons to the receptor, while LUMO represents the nucleophilic attack site by calculating its ability to accept electrons from the receptor (51).

**Table 3:** Frontier molecular orbital energy of the selected five compounds.

Compounds	HOMO (eV)	LUMO (eV)	Energy gap (eV)	Electronic chemical potential (μ) (eV)
STOCK1N-62634	−0.169	−0.088	0.081	−0.04
STOCK1N-96109	−0.170	−0.063	0.107	−0.05
STOCK1N-94672	−0.176	−0.050	0.126	−0.06
STOCK1N-89819	−0.205	−0.103	0.102	−0.05
STOCK1N-80570	−0.201	−0.078	0.123	−0.06

**Figure 3:**
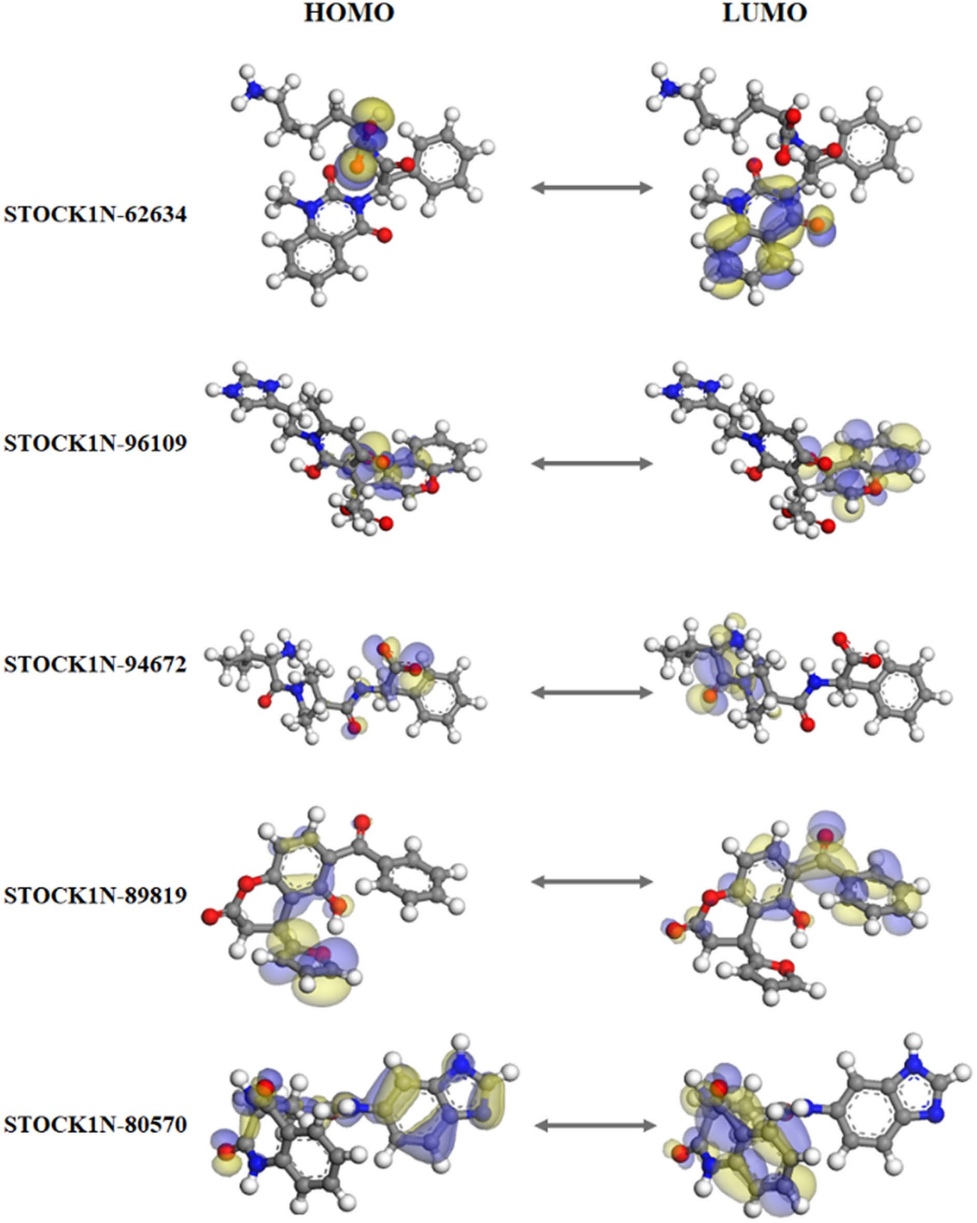
Frontier molecular orbital plot of HOMO and LUMO. Blue and yellow isosurfaces illustrate the electron build-up region and electron loss regions, respectively.

Figure 3 represents the molecular orbital plot of the selected compounds in which the location of the molecules can be identified from HOMO, LUMO energy values, that is represented in Table 3. Blue and yellow isosurfaces exhibit electron build-up region and electron loss regions, respectively. The HOMO energy values range from −0.205 eV in STOCK1N-89819 to −0.169 eV in STOCK1N-62634, while the LUMO energy values ranged from −0.103 eV in STOCK1N-89819 to −0.050 in STOCK1N-94672. The energy gap (HOMO/LUMO Gap) is the energy difference between the LUMO and HOMO levels, which is inversely proportional to the stabilizing energy of the molecule. The calculated energy gap values of all the selected compounds varied from 0.081 eV to 0.126 eV. Electronic chemical potential (μ) is the total amount of energy required to remove one single electron from the molecule (51). The μ values range from −0.06 eV in STOCK1N-94672 and STOCK1N-80570 to −0.04 eV in STOCK1N-62634. Low values of μ in all the five compounds suggest strong binding affinities. Low energy gap values and negative HOMO and LUMO values of all the selected compounds suggest their ability to donate and accept electrons with the receptor for a strong protein–ligand interaction and, thus, high inhibition of 3C protease protein.

### Molecular dynamics simulation analysis

The structural stability and dynamic behaviors of all the top five screened compounds with 3C protease protein was investigated through the 300 ns MD simulation study.

#### Structural deviation analysis through RMSD

**Figure 4:**
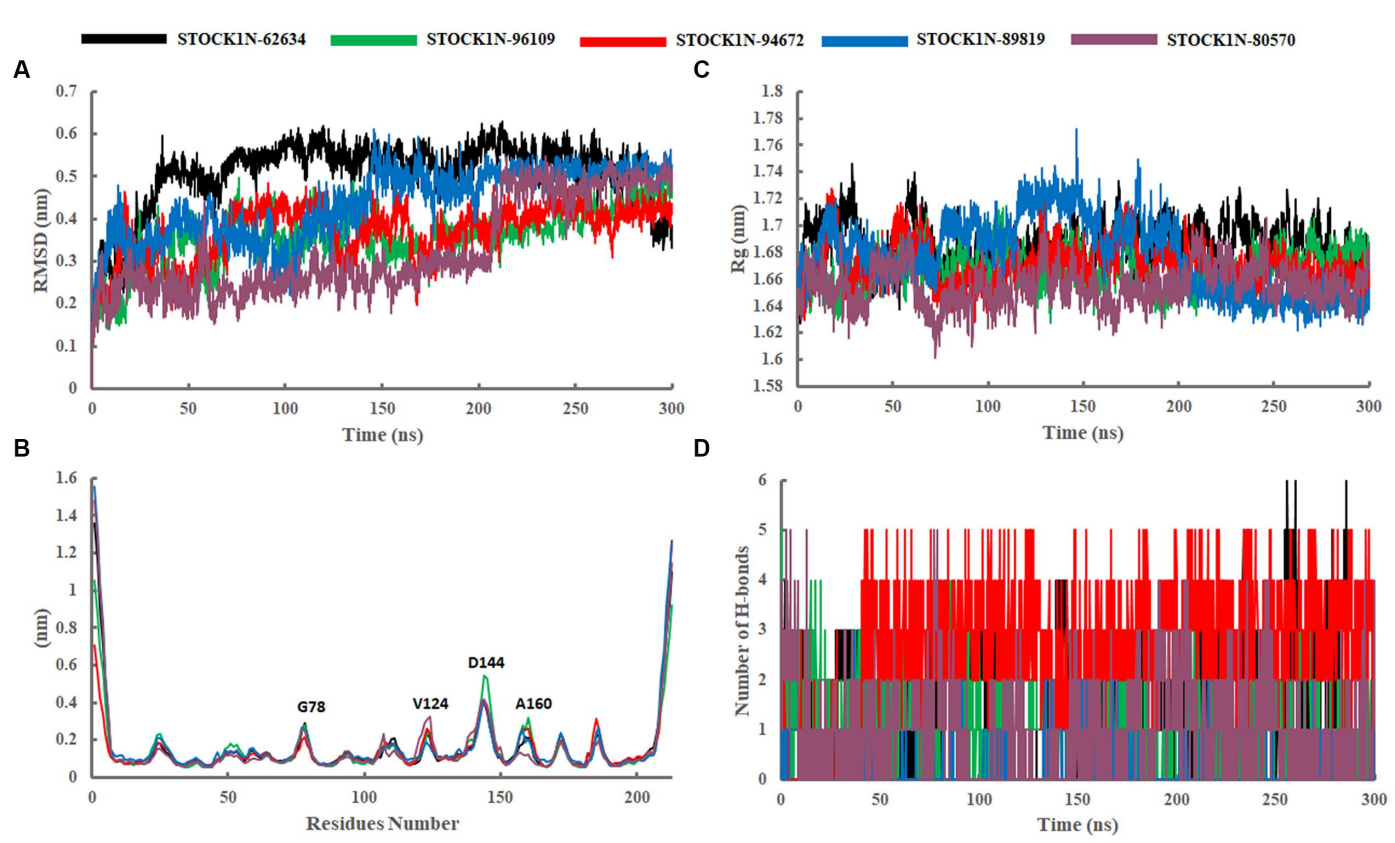
Stability analysis through 300 ns MD simulation trajectory. **(A)** RMSD of backbone atoms. **(B)** Residual flexibility analysis through RMSF. **(C)** Compactness analysis through Rg. **(D)** Changes in the number of H-bonds throughout the simulation. Black, green, red, blue, and purple color represent the complexes with 3C protease and compounds STOCK1N-62634, STOCK1N-96109, STOCK1N-94672, STOCK1N-89819, and STOCK1N-80570, respectively.

The structural stability of all five screened complexes were analyzed through RMSD of the backbone atoms (C, Cα, and N) from 300 ns MD simulation trajectory. The RMSD graph of the protein backbone with reference to the initial conformation with ligands STOCK1N-62634, STOCK1N-96109, STOCK1N-94672, STOCK1N-89819, and STOCK1N-80570 exhibited an average RMSD of 0.51 nm, 0.36 nm, 0.37 nm, 0.44 nm, and 0.33 nm, respectively (Figure 4A). With all the five compounds, the 3C protease protein is getting stable toward the last 250 ns of MD simulation. These data also suggest that the compounds STOCK1N-96109, STOCK1N-94672, and STOCK1N-89819 are highly stable complexes relative to others. Moreover, the dynamic behaviors of all five compounds were studied by calculating the RMSD of each ligand, and the result revealed that compounds STOCK1N-62634, STOCK1N-96109, STOCK1N-94672, STOCK1N-89819, and STOCK1N-80570 exhibited an average RMSD of 0.29 nm, 0.18 nm, 0.24 nm, 0.17 nm, and 0.18 nm, respectively (Supplementary Figure S3A). In particular, compounds STOCK1N-94672 and STOCK1N-96109 exhibited higher ligand stability than other compounds.

#### Residual flexibility analysis through RMSF

The fluctuation of each amino acid in 3C protease protein during the MD simulation was analyzed through RMSF and shown in Figure 4B. The receptor 3C protease after binding to compounds STOCK1N-62634, STOCK1N-96109, STOCK1N-94672, STOCK1N-89819, and STOCK1N-80570 exhibited an average RMSF of 0.16 nm, 0.15 nm, 0.15 nm, 0.17 nm, and 0.16 nm, respectively. Interestingly, the residual fluctuations are almost similar for all five screened compounds. After binding to all five compounds, the active site and catalytic triad residues such as C142, D84, C163, and H46 have been revealed to be stable throughout the 300 ns trajectory. G78, V124, D144, and A160 of 3C protease exhibited higher fluctuations after binding to ligands.

#### Compactness analysis through Rg

The compactness of the receptor after binding to ligands was analyzed through Rg. It is the average distance of each atom in the molecule from the center of mass, which provides insights into the overall spatial distribution of atoms within the molecule. The Rg calculation revealed an average value of 1.69 nm, 1.67 nm, 1.67 nm, 1.68 nm, and 1.66 nm for the complexes with compounds STOCK1N-62634, STOCK1N-96109, STOCK1N-94672, STOCK1N-89819, and STOCK1N-80570, respectively (Figure 4C). Almost all the five compounds are getting stable toward the last 150 ns of simulation. In particular, the complexes with STOCK1N-96109 showed a more stable and compact conformations as compared to others.

#### Solvent accessible surface area analysis

The SASA of all five complexes was computed throughout the simulation and represented in Supplementary Figure S3B. The SASA results revealed an average value of 121.74nm^2^, 119.73 nm^2^, 119.29 nm^2^, 119.82 nm^2^, and 118.97 nm^2^ for receptor 3C protease with ligands STOCK1N-62634, STOCK1N-96109, STOCK1N-94672, STOCK1N-89819, and STOCK1N-80570, respectively. All five complexes exhibited similar kinds of SASA values with an average value of 119 nm^2^, revealing minimal changes after the binding of each compound.

#### Interaction analysis through hydrogen bonding

The stability of all five complexes and protein–ligand interactions were analyzed through the number of hydrogen bonds between them throughout the 300 ns MD simulations and represented in Figure 4D. Compounds STOCK1N-62634 and STOCK1N-96109 exhibited an average of 0–2 number of H-bonds throughout the simulation, while compounds STOCK1N-89819 and STOCK1N-80570 exhibited an average of 0–1 number of H-bonds. Compound STOCK1N-94672 showed the highest number of H-bonds interacting with the receptor (average of 2–3) as compared to others.

#### Principal component analysis-based free energy landscape

**Figure 5:**
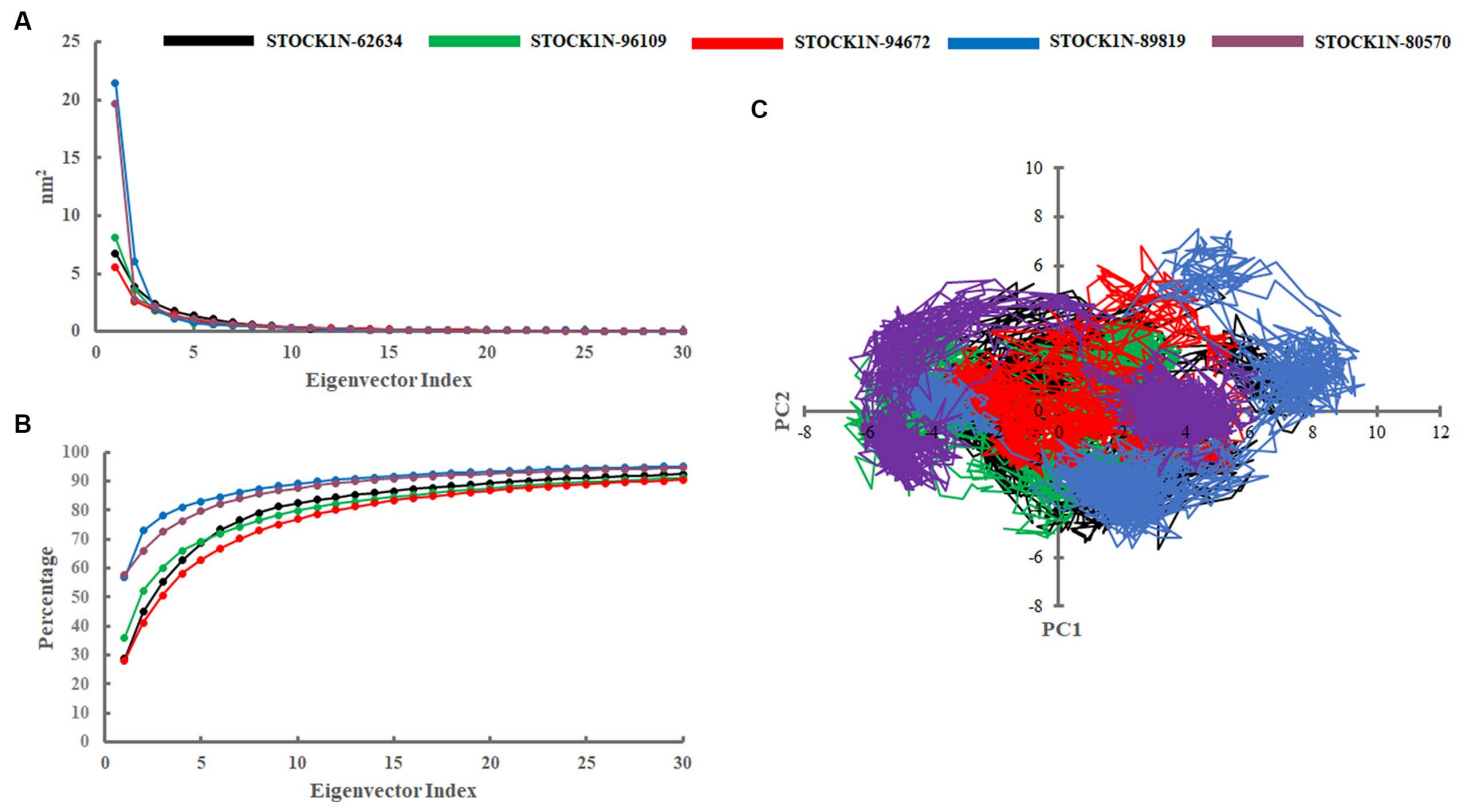
Principal component analysis. **(A)** Eigenvalues derived from the last 200 ns simulation. **(B)** Cumulative percentage of first 30 eigenvectors. **(C)** Projection of first two principal components (PC1 and PC2) for all five complexes. Black, green, red, blue, and purple color represent complexes with 3C protease and compounds STOCK1N-62634, STOCK1N-96109, STOCK1N-94672, STOCK1N-89819, and STOCK1N-80570, respectively.

**Figure 6:**
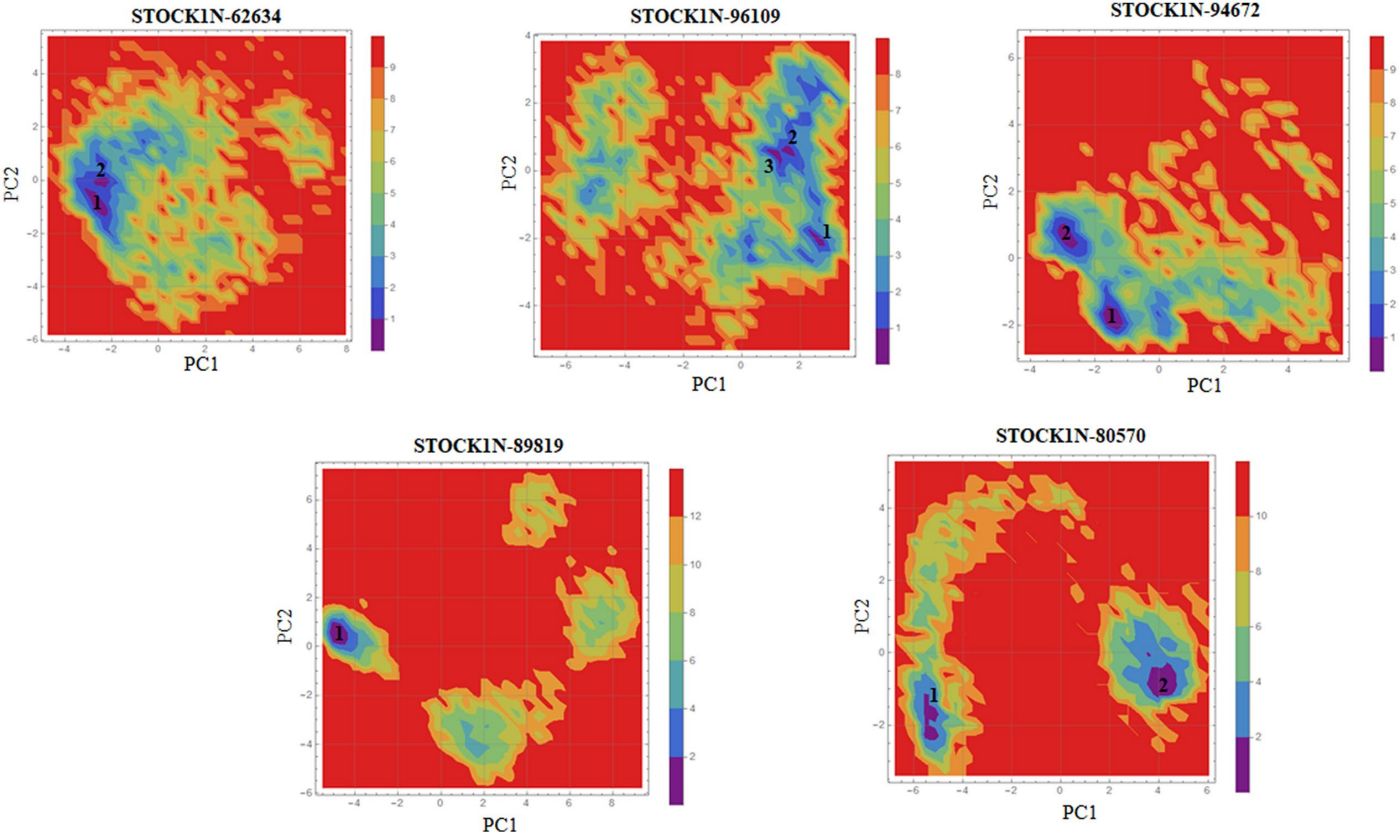
Gibbs’ free energy landscape using PC1 and PC2. The lower energy systems were illustrated in deep blue color.

Principal component analysis was performed to understand the global concentrated motion of all five complexes after ligand binding. This method can help to identify the most significant conformational changes that take place during the simulation. The first 30 eigenvectors were considered to calculate the eigenvalues and percentage changes from the last 200 ns MD simulation (Figure 5A). The first two PCs exhibited larger motions as compared to others with a cumulative variance of 44.97, 52.16, 41.20, 72.88, and 66.14% for STOCK1N-62634, STOCK1N-96109, STOCK1N-94672, STOCK1N-89819, and STOCK1N-80570, respectively (Figure 5B). Furthermore, a 2D plot was generated by assessing PC1 and PC2 for all five complexes and is represented in Figure 5C. Complexes with compounds STOCK1N-62634, STOCK1N-96109, and STOCK1N-94672 showed less overall distribution of protein conformation as compared to others. We have also analyzed the Gibbs free energy landscape (FEL) using the first two PCs in order to visualize the transformation that takes place during MD simulation where deep blue color represents the global minima (Figure 6). FEL can accurately depict the most stable conformational ensembles by analyzing the structural changes in protein–ligand complexes. The receptor with compounds STOCK1N-62634 and STOCK1N-94672 exhibited one major cluster with two global minima, while others exhibited more than one cluster. Compound STOCK1N-89819 and STOCK1N-80570 exhibited dispersed clusters with one and two global minima, respectively. In addition, we have also generated the low energy conformations from all the global minima and aligned to their respective initial conformation (Supplementary Figure S4). Structural alignment results revealed that all the low-energy conformations except the complexes with compound STOCK1N-94672 are observed to be in the binding pocket of 3C protease.

### Binding free energy analysis

**Figure 7:**
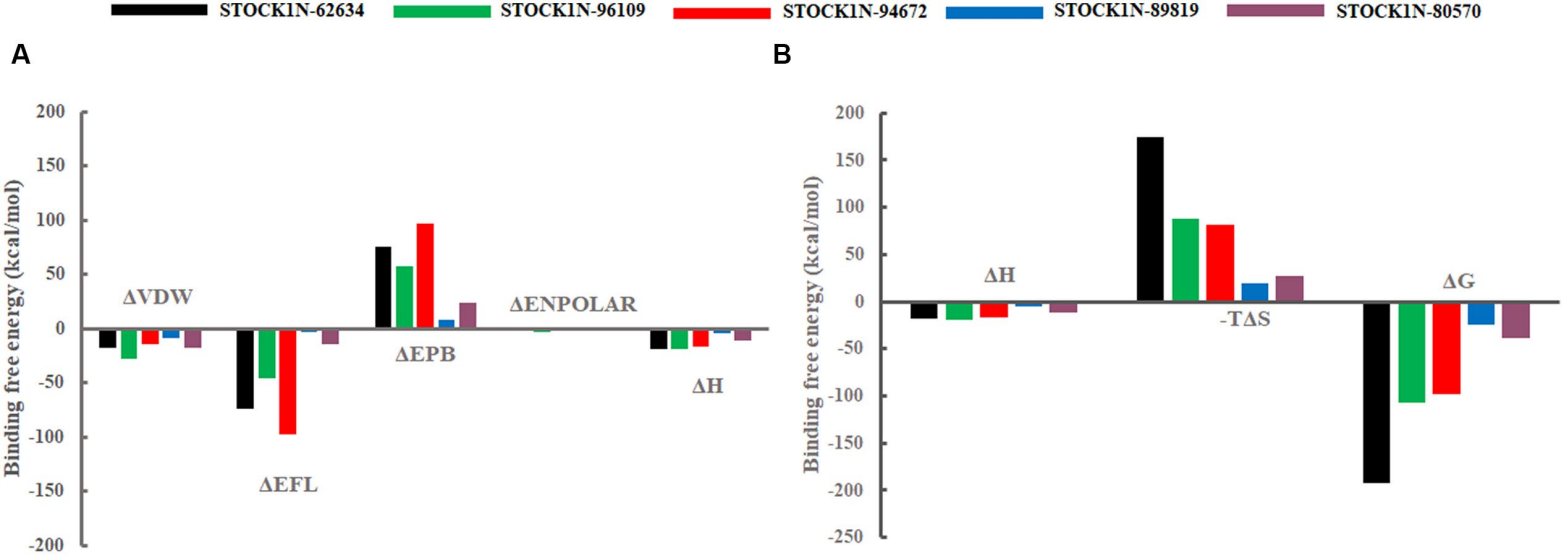
Free energy components in kcal/mol. **(A)** Binding energy, ΔH is the summation of ΔVDW, ΔEFL, ΔEPB, and ΔENPOLAR. **(B)** Total energy calculated from the binding energy and entropy components, i.e., ΔG = ΔH-TΔS. Black, green, red, blue, and purple color represent complexes with 3C protease and compounds STOCK1N-62634, STOCK1N-96109, STOCK1N-94672, STOCK1N-89819, and STOCK1N-80570, respectively.

The binding affinity of each ligand with the receptor was calculated from the last 200 ns of MD simulation using molecular mechanics/Poisson–Boltzmann surface area (MM-PBSA) approach. In this study, we have calculated the van der Waals energy (ΔVDW), electrostatic energy (ΔEFL), electrostatic contribution to the solvation free energy calculated by the Poisson–Boltzmann model (ΔEPB), non-polar contributions (ΔENPOLAR), and entropy (TΔS) of each complex and mentioned it in Table 4 and Figure 7. Compound STOCK1N-62634 showed the highest binding affinity of −192.25 kcal/mol with the receptor followed by compounds STOCK1N-96109 (−106.85 kcal/mol), STOCK1N-94672 (−97.86 kcal/mol), STOCK1N-89819 (−24.23 kcal/mol), and STOCK1N-80570 (−38.32 kcal/mol). According to the binding free energy analysis, electrostatic energy components (ΔEFL) and van der Waals energy components (ΔVDW) contribute significantly to the binding of these natural compounds.

**Table 4:** MM-PBSA based average binding free energy of 3C protease with each compound in kcal/mol.

Compounds	ΔVDW	ΔEFL	ΔEPB	ΔENPOLAR	ΔH	-TΔS	ΔG
STOCK1N-62634	−17.30 ± 0.82	−73.54 ± 1.80	75.03 ± 1.20	−2.49 ± 0.07	−18.29 ± 2.54	173.96 ± 0.05	−192.25 ± 8.78
STOCK1N-96109	−27.98 ± 0.69	−45.27 ±4.24	57.75 ±1.69	−3.54 ±0.02	−19.04 ±4.69	87.81 ±0.05	−106.85 ±5.20
STOCK1N-94672	−14.04 ±1.26	−97.04 ±5.82	97.08 ±3.48	−2.18 ±0.05	−16.17 ±6.98	81.69 ±0.05	−97.86 ±4.36
STOCK1N-89819	−8.29 ±1.09	−3.38 ±2.56	8.16 ±0.91	−1.17 ±0.08	−4.68 ±3.05	19.55 ±0.05	−24.23 ±4.69
STOCK1N-80570	−17.78 ±0.33	−14.66 ±3.52	23.56 ±1.50	−2.12 ±0.06	−11.00 ±3.95	27.32 ±4.80	−38.32 ±7.93

**Figure 8:**
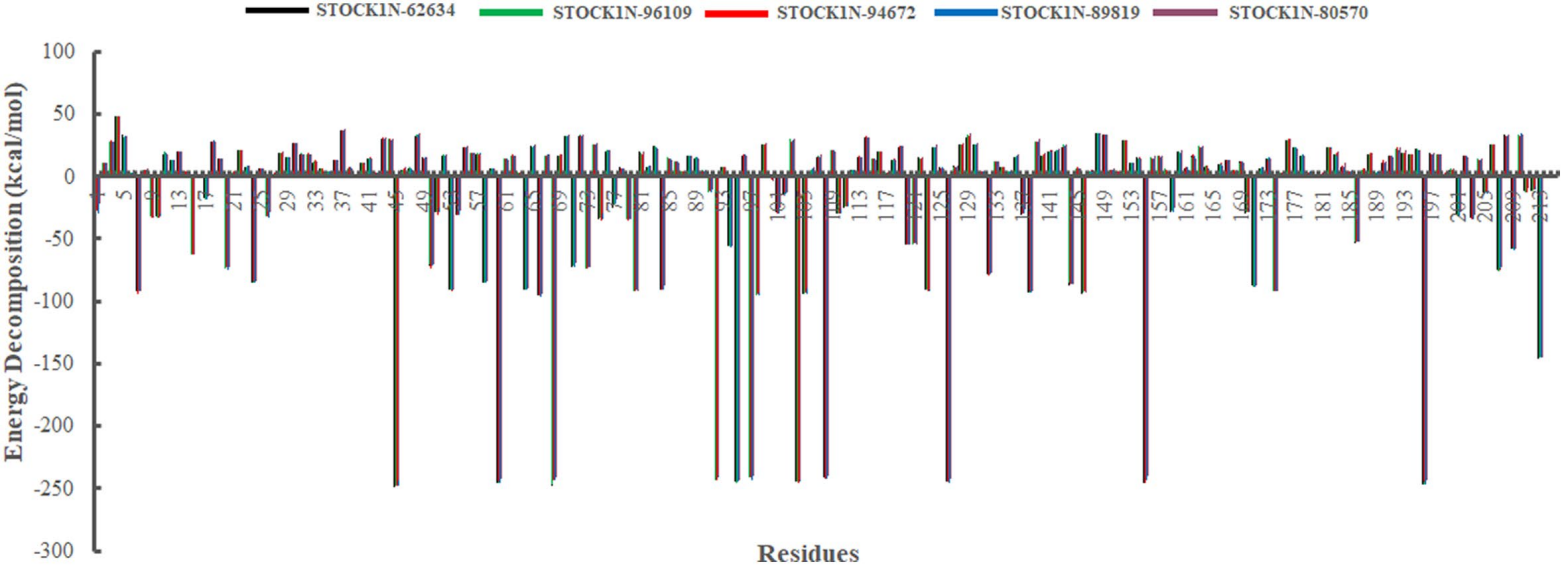
Energy decomposition of each amino acid in 3C protease upon ligand binding. Black, green, red, blue, and purple color represent complexes with 3C protease and compounds STOCK1N-62634, STOCK1N-96109, STOCK1N-94672, STOCK1N-89819, and STOCK1N-80570, respectively.

In addition, we have also analyzed the residual binding energy of each amino acid in the receptor upon ligand binding. This is a key method for determining the hotspot residues that play a major role in the ligand binding during simulation. Energy decomposition results revealed that most of the arginine residues such as R45, R60, R68, R92, R95, R97, R104, R108, R126, R155, and R196 contribute significantly to the ligand binding, with an average energy of −240 kcal/mol (Figure 8). Among active sites and catalytic residues, D84 contributed significantly with an average energy of −90 kcal/mol for all complexes. Residues such as D7, D24, D53, D66, D80, D98, D103, D174, and E213 also played a major role in energy contribution.

## Discussion

FMDV is a highly infectious ssRNA virus that affects many species such as cattle, sheep, goat, and pig, significantly affecting the global livestock economy. Because of its high mutation rate and contagious nature, it infects more than 70 species of animals and has become a major health concern in livestock industries (1). Several nations encountered severe FMD outbreaks that affected the growth of animal husbandry and foreign trade, negatively impacting the global economy (52). FMDV outbreaks can lead to substantial direct economic losses for the livestock industry. These losses primarily stem from animal deaths, decreased productivity, and trade restrictions imposed to control the spread of the disease. As per a research study published in the journal Transboundary and Emerging Diseases, FMDV outbreaks in the United Kingdom between 2001 and 2010 resulted in direct costs ranging from £8 million (approximately $10 million) to £180 million (approximately $225 million) per outbreak (53). It can also lead to trade disruptions, with many countries imposing restrictions on the imports and exports of livestock and meat products from the affected regions. These disruptions can significantly impact the livestock industry’s ability to export and earn revenue. For example, during the FMDV outbreak in the United Kingdom in 2001, the country banned exports of live animals, meat, and dairy products, causing estimated losses of approximately £2.4 billion (approximately $3 billion) to the industry. Different countries adopted several vaccination strategies based on prevailing FMDV strains, but vaccination does not cure the infection and takes several weeks to elicit an immune response. Since no specific antiviral treatments are available for FMD, there is a need to explore new and effective antiviral drugs against FMDV. It is important to highlight that losses incurred by the livestock industry due to FMDV outbreaks can have severe consequences for livelihoods, animal welfare, food security, and the overall economy of affected regions or countries. By quantifying these losses, it highlights the urgency of developing effective antiviral treatments to combat FMDV, reduce the impact of outbreaks, and protect the livestock industry worldwide. The involvement of 3C protease in FMDV replication and proteolytic activity recommends it as one of the critical targets for antiviral drug development (10, 11). Research on natural compounds as antiviral medications has drawn much attention due to their low toxicity, broad-spectrum activity, and diverse mechanisms of action. However, exploring FMDV inhibitors from natural resources is challenging because the extraction of these compounds and the evaluation of antiviral activity demand high funding and specialized experimental equipment (14). The current study uses computational approaches to explore potential natural compounds from an extensive natural compounds database against 3C protease through structure-based virtual screening followed by MD simulation analysis of the top five screened compounds.

The natural product dataset from the InterBioScreen database, having 69,040 compounds, was retrieved and considered for structure-based virtual screening. Furthermore, the top five screened compounds having high binding affinity and interaction with crucial residues were considered for further analysis. The SBVS results revealed that compound STOCK1N-62634 exhibited the highest binding affinity of −9.575 kcal/mol by forming four hydrogen bonds with H46, D146, D144, and G184. Moreover, residues such as M143 and A183 were involved in pi-alkyl interaction with the compound STOCK1N-62634. STOCK1N-96109 interacts with 3C protease by forming four hydrogen bond interactions at E50, D146, M143, and G184 with a binding affinity of −7.878 kcal/mol. Additionally, it formed one pi-cation interaction with H46 and alkyl bonds with C163, A183, and A160. Compounds STOCK1N-94672 and STOCK1N-89819 exhibited the similar binding affinity of −7.674 kcal/mol and − 7.451 kcal/mol, respectively, by forming two hydrogen bonds and one pi-alkyl interaction, each with A160. Compound STOCK1N-94672 formed two H-bonds with H46 and G161, while compound STOCK1N-89819 formed two H-bonds with M143 and G184. Compound STOCK1N-80570 showed a docking score of −7.34 kcal/mol by forming five H-bonds with M143, H181, G184, N186, and Y190 and one pi-alkyl interaction with A160. Mostly, all five compounds formed at least one pi-alkyl interaction with A160. These non-covalent interactions are crucial in medicinal chemistry for designing drugs with high affinity, selectivity, and favorable pharmacokinetic properties. It enables medicinal chemists to optimize the interactions between drugs and their target proteins, ultimately developing effective and safe pharmaceutical agents. According to one of the recent studies by Theerawatanasirikul et al. (54), a similar attempt to identify small compounds against 3C protease and revealed the binding affinity of top hit compounds in the range from −6.6 to −6.8 kcal/mol. Most computational chemists focus on the structure or reactivity of the ligands, including geometry optimization of the molecule as a main component. According to the frontier molecular orbital (FMO) theory of chemical reactivity, the contact between HOMO and LUMO of the reacting species results in the transition of one electron. HOMO values explain the ability to donate electrons, while LUMO explains the ability to accept electrons from the receptor (55). Hence, geometry optimization of the selected five compounds was performed through DFT calculation, and all of them exhibited spatial arrangements of electron density in the HOMO that disperse across the covalent bonds formed between adjacent p orbital overlaps, whereas the electron density in the LUMO was dispersed across the aromatic rings. The negative HOMO and LUMO energy values derived from DFT calculation indicate their strong electron-accepting and electron-donating potentials. Moreover, the small energy gap values in all the compounds indicate the movement of electrons from the HOMO to LUMO, resulting a solid binding interaction with the receptor protein. Furthermore, we have also predicted the physiochemical properties of all five screened compounds, and all of them revealed good drug-like behavior without violating Lipinski’s rule of five. Therefore, a 300 ns MD simulation analysis was performed for 3C protease with all five screened compounds, i.e., STOCK1N-62634, STOCK1N-96109, STOCK1N-94672, STOCK1N-89819, and STOCK1N-80570, for a detailed structural insight and dynamic behavior of protein and protein–ligand complexes.

The dynamic behavior of all five complexes was analyzed through several structural parameters such as RMSD, RMSF, Rg, SASA, and H-bonds. The conformation stability of backbone atoms was calculated through RMSD analysis, and the results revealed that, except for STOCK1N-80570, all the four complexes exhibited stable conformation throughout the trajectory, but compound STOCK1N-80570 exhibited a slight fluctuation after 200 ns. However, it again gets stabilized toward the end. The conformational compactness of all complexes was also calculated through the Rg. The lower the Rg values, the higher the compactness. The calculated Rg values over the 300 ns MD simulation time scale showed overall compact and stable conformations of all five complexes. Moreover, all five complexes exhibited lower Rg values toward the last 200 ns of simulation, resulting in more compact and structural stability. In addition, we have calculated the RMSF, SASA, and H-bonds throughout the 300 ns MD trajectory, and interestingly, no residual fluctuations were observed in the active site and catalytic residues of 3C protease after ligand binding. The SASA analysis also supports the RMSD and Rg analysis by suggesting stable and minimal changes after ligand binding. All five screened compounds’ interaction with 3C protease was analyzed through H-bond analysis throughout the trajectory, and the results indicate that compound STOCK1N-94672 exhibited the highest average number of H-bonds with the receptor. In comparison, others formed at least one H-bond with the receptor throughout the trajectory, which indicates a strong interaction between all five natural compounds and the receptor. The global dynamic behavior of all complexes was analyzed through PCA and Gibb’s free energy landscape (FEL), and the results derived from the first two PCs indicate that 3C protease with all five compounds exhibited fewer dynamics. Mainly, compounds STOCK1N-96109 and STOCK1N-94672 exhibited less overall relative motion than others. Furthermore, we have calculated the binding free energy and each residual binding energy using the MM-PBSA method from the last 200 ns of MD simulations. MM-PBSA is a widely used approach to calculate the free energy in biomolecular complexes such as the protein–ligand complex, protein–DNA complex, or protein–protein complex (47, 49). MM-PBSA calculations are commonly used in computer-aided drug design and lead optimization studies to evaluate the binding affinities of ligands, compare different ligand poses, and guide the selection of promising drug candidates. MM-PBSA analysis suggests that compound STOCK1N-62634 showed the highest binding affinity of −192.25 kcal/mol with the receptor, followed by STOCK1N-96109 (−106.85 kcal/mol), STOCK1N-94672 (−97.86 kcal/mol), STOCK1N-89819 (−24.23 kcal/mol), and STOCK1N-80570 (−38.32 kcal/mol), and these natural compounds, particularly STOCK1N-62634, STOCK1N-96109, and STOCK1N-94672, can be considered as a potential lead for the inhibition of 3C protease (48, 56). From the above results, we believe that these natural compounds could be potential antiviral inhibitors for the 3C protease of FMDV.

## Conclusion

Foot-and-mouth disease virus (FMDV) is a highly contagious virus that infects many species such as cattle, sheep, goats, and pigs and has become a significant health concern in livestock industries. Currently, no particular antiviral treatment is available for FMD, making exploring new inhibitors against FMDV critically important. Many countries adopted different vaccine strategies based on the prevailing FMDV strains, but it is hard to cure the infection, and it takes several days to elicit an immune response with the vaccine. Given the importance of 3C protease in viral replication and pathogenesis, we employed a structure-based virtual screening and MD simulation approach to explore potential inhibitors from a large natural compound dataset. The integration of computer-based drug discovery (CBDD) and MD simulations in drug discovery for viral diseases holds the potential to revolutionize the field by enabling more efficient, targeted, and personalized approaches to drug development. These technologies contribute to a better understanding of the underlying biology, accelerate the identification of potential drug candidates, and improve the overall success rate of drug discovery pipelines. The results identified five potential lead antiviral inhibitors, particularly STOCK1N-62634, STOCK1N-96109, and STOCK1N-94672, with effective drug-likeness properties, which can be further evaluated using clinical and experimental approaches for future management of FMDV infection.

## Data availability statement

The original contributions presented in the study are included in the article/Supplementary material, further inquiries can be directed to the corresponding authors.

## Author contributions

SS: Conceptualization, Formal analysis, Investigation, Writing – original draft. H-KL: Funding acquisition, Supervision, Writing – review & editing. DS: Conceptualization, Formal analysis, Funding acquisition, Supervision, Writing – review & editing.

## Footnotes

^1^ http://www.rcsb.org

^2^ https://www.uniprot.org/

^3^ https://www.ibscreen.com
