# Supplementary material for: Structure-based virtual screening and molecular dynamics studies to explore potential natural inhibitors against 3C protease of foot-and-mouth disease virus
Source: Front Vet Sci. 2024 Jan 17;10:1340126. doi: 10.3389/fvets.2023.1340126 (PMC10827980; doi:10.3389/fvets.2023.1340126)
Supplement: Supplementary file 1 [file Data_Sheet_1.docx]

**Supplementary Information**

**Structure-based virtual screening and molecular dynamics studies to explore potential natural inhibitors against 3C protease of foot-and-mouth disease virus.**

Sthitaprajna Sahoo^1^, Hak-Kyo Lee^1,2*^, Donghyun Shin^1^*

*^1^ Department of Agricultural Convergence Technology, Jeonbuk National University, Jeonju 54896, Republic of Korea*

*^2^ Department of Animal Biotechnology, Jeonbuk National University, Jeonju 54896, Republic of Korea*

*^*^Correspondence:* [*breedlee@jbnu.ac.kr*](mailto:breedlee@jbnu.ac.kr) *(H.L.) and* [*sdh1214@gmail.com*](mailto:sdh1214@gmail.com) *(D.S.)*


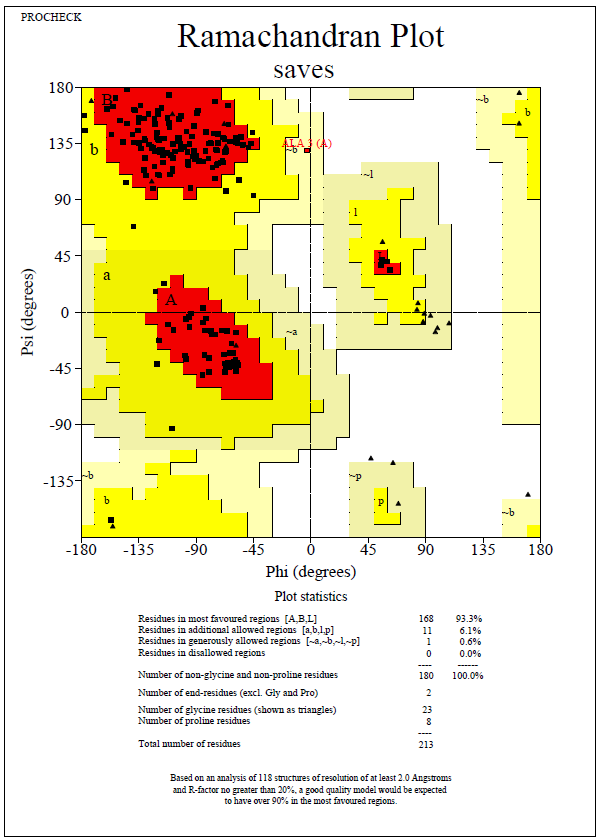


Figure S1: Validation of AlphaFold2 modeled structure of FMDV 3C protease using Ramachandran plot.


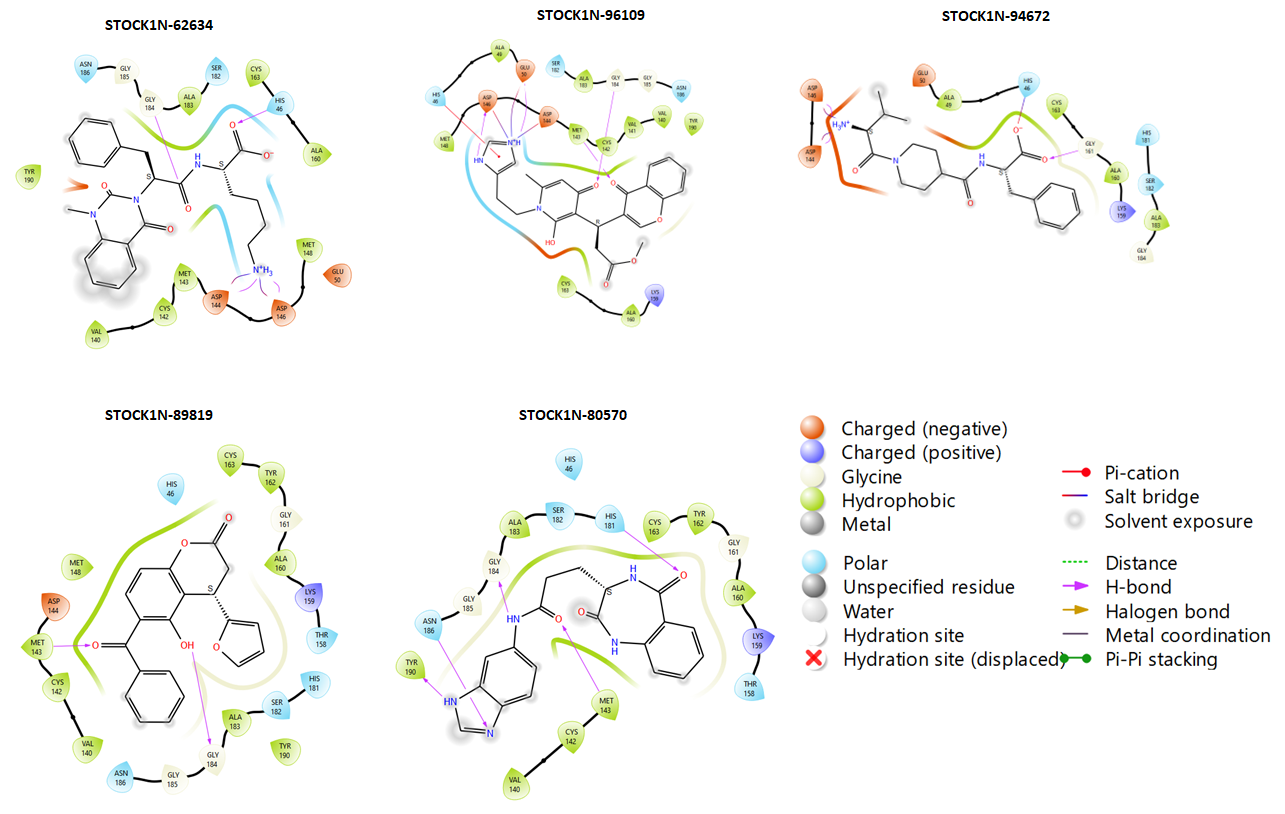


Figure S2: 2D molecular interactions of all the five screened natural compounds with 3C protease protein.


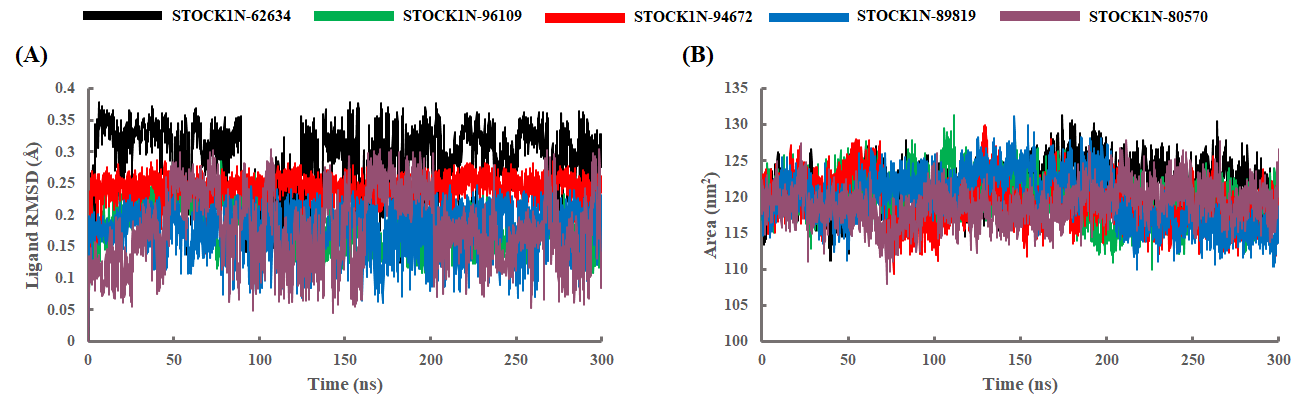


Figure S3: (A) RMSD of the ligand through 300ns MD simulation. (B) SASA (solvent accessible surface area) of all complexes throughout the simulation. Black, green, red, blue, and purple color represents complex with 3C protease and compounds STOCK1N-62634, STOCK1N-96109, STOCK1N-94672, STOCK1N-89819, and STOCK1N-80570 respectively.


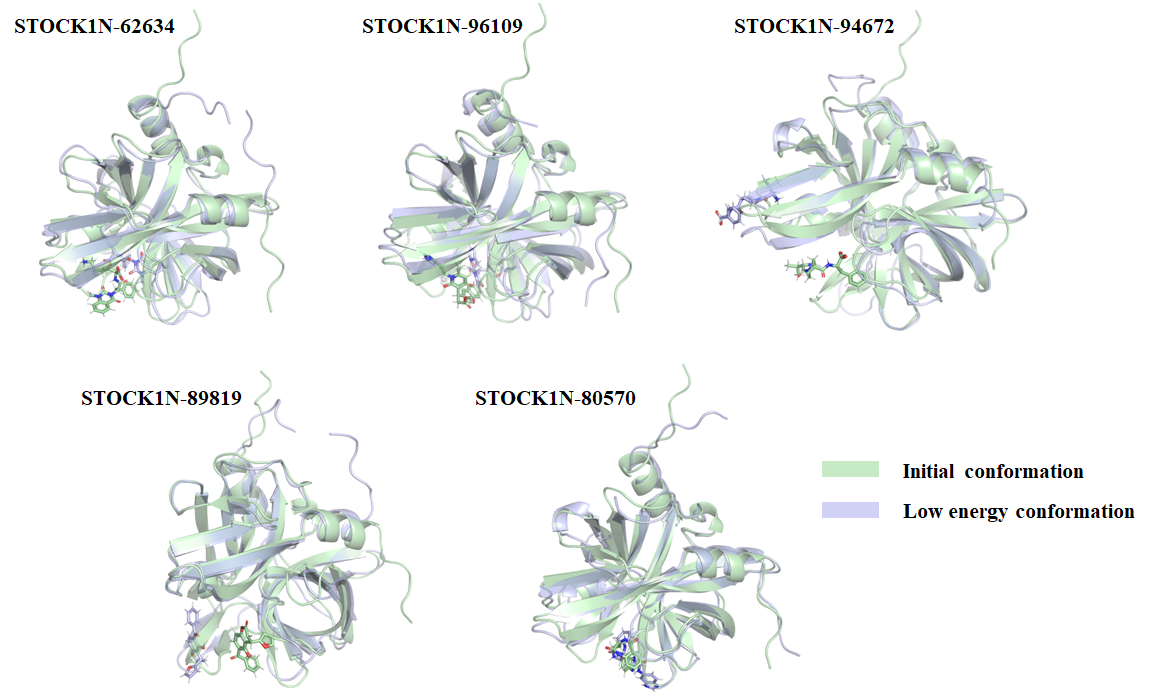


Figure S4: Structural alignment of all five complexes' initial and low energy conformations. Pale green and light blue colors represent initial and low energy conformations respectively.
